# Hypertension, Microvascular Pathology, and Prognosis After an Acute Myocardial Infarction

**DOI:** 10.1161/HYPERTENSIONAHA.117.10786

**Published:** 2018-07-16

**Authors:** David Carrick, Caroline Haig, Annette M. Maznyczka, Jaclyn Carberry, Kenneth Mangion, Nadeem Ahmed, Vannesa Teng Yue May, Margaret McEntegart, Mark C. Petrie, Hany Eteiba, Mitchell Lindsay, Stuart Hood, Stuart Watkins, Andrew Davie, Ahmed Mahrous, Ify Mordi, Ian Ford, Aleksandra Radjenovic, Paul Welsh, Naveed Sattar, Kirsty Wetherall, Keith G. Oldroyd, Colin Berry

**Affiliations:** 1 From the British Heart Foundation Glasgow Cardiovascular Research Centre, Institute of Cardiovascular and Medical Sciences, University of Glasgow, United Kingdom (D.C., A.M.M., J.C., K.M., N.A., V.T.Y.M., M.M., M.C.P., I.M., A.R., P.W., N.S., K.G.O., C.B.); 2 West of Scotland Heart and Lung Centre, Golden Jubilee National Hospital, Clydebank, United Kingdom (D.C., A.M.M., J.C., K.M., N.A., V.T.Y.M., M.M., M.C.P., H.E., M.L., S.H., S.W., A.D., A.M., I.M., K.G.O., C.B.); 3 Robertson Centre for Biostatistics, University of Glasgow, United Kingdom (C.H., I.F., K.W.).

**Keywords:** atherosclerosis, hypertension, myocardial infarction, prognosis, reperfusion injury

## Abstract

The rationale for our study was to investigate the pathophysiology of microvascular injury in patients with acute ST-segment–elevation myocardial infarction in relation to a history of hypertension. We undertook a cohort study using invasive and noninvasive measures of microvascular injury, cardiac magnetic resonance imaging at 2 days and 6 months, and assessed health outcomes in the longer term. Three hundred twenty-four patients with acute myocardial infarction (mean age, 59 [12] years; blood pressure, 135 [25] / 79 [14] mm Hg; 237 [73%] male, 105 [32%] with antecedent hypertension) were prospectively enrolled during emergency percutaneous coronary intervention. Compared with patients without antecedent hypertension, patients with hypertension were older (63 [12] years versus 57 [11] years; *P*<0.001) and a lower proportion were cigarette smokers (52 [50%] versus 144 [66%]; *P*=0.007). Coronary blood flow, microvascular resistance within the culprit artery, infarct pathologies, inflammation (C-reactive protein and interleukin-6) were not associated with hypertension. Compared with patients without antecedent hypertension, patients with hypertension had less improvement in left ventricular ejection fraction at 6 months from baseline (5.3 [8.2]% versus 7.4 [7.6]%; *P*=0.040). Antecedent hypertension was a multivariable associate of incident myocardial hemorrhage 2-day post-MI (1.81 [0.98–3.34]; *P*=0.059) and all-cause death or heart failure (n=47 events, n=24 with hypertension; 2.53 [1.28–4.98]; *P*=0.007) postdischarge (median follow-up 4 years). Severe progressive microvascular injury is implicated in the pathophysiology and prognosis of patients with a history of hypertension and acute myocardial infarction.

**Clinical Trial Registration—:**

URL: http://www.clinicaltrials.gov. Unique identifier: NCT02072850.

## Abstract 2

Supplemental Digital Content is available in the text.

Hypertension has a continuous, age-related risk of mortality from ischemic heart disease.^1^ At least 30% of adults have a history of hypertension in developed countries,^2,3^ and hypertension is independently associated with adverse cardiac outcome after acute myocardial infarction (MI).^4–11^ However, the mechanisms for this association are unclear. Patients who present with acute ST-segment–elevation myocardial infarction (STEMI) and a history of hypertension are older and generally have a higher burden of risk factors,^11,12^ except for cigarette smoking which associates with male sex and younger age.^11,12^ The size of infarction is a key determinant of survival post-MI, but previous studies^11,12^ have not found any association between hypertension status and infarct size. Therefore, the mechanisms underlying the association between hypertension status and health outcomes post-MI remain unclear.

Hypertension is a risk factor for coronary heart disease.^13^ The pathophysiology includes left ventricular hypertrophy,^14^ coronary endothelial dysfunction,^15^ accelerated coronary atherosclerosis,^16,17^ abnormal coronary artery remodeling,^18^ coronary microvascular dysfunction,^19^ and epicardial fat.^20^ Accordingly, preexisting coronary heart disease may predispose patients with antecedent hypertension to enhanced myocardial reperfusion injury. Severe microvascular injury within the infarct zone manifests acutely as microvascular obstruction affecting about half of all-comers with STEMI,^21,22^ and subsequently resolving in half of these patients by 10 days.^22^ In patients with persistent microvascular obstruction, progressive irreversible capillary degradation occurs leading to infarct zone hemorrhage, which is an independent predictor of death or heart failure in the longer term.^21,22^ To date, the associations between antecedent hypertension and microvascular injury post-MI are unclear.

We investigated the natural history of hypertension status, microvascular pathology, and prognosis in all-comers with acute STEMI. We measured microvascular function directly in the culprit coronary artery acutely using a sensor mounted on an intracoronary guidewire and noninvasively using the surface ECG. We subsequently used multiparametric cardiac magnetic resonance (CMR) to assess the evolution of infarct pathologies and left ventricular function and volumes at 2-day and 6-month post-MI.

We hypothesized that a history of hypertension would be associated with enhanced microvascular dysfunction within the culprit coronary artery acutely, and more abundant microvascular pathologies, including microvascular obstruction and myocardial hemorrhage, independent of the size of infarction, when assessed using CMR 2 days later. This hypothesis implicates microvascular damage within the infarct zone as an underpinning mechanism leading to less myocardial salvage, greater adverse left ventricular remodeling, and an increased risk of heart failure and death after an acute MI.

## Methods

The data that support the findings of this study can be requested from the following URL: http://www.CORportal.net.

### Study Population

We performed a prospective cohort study in a regional cardiac center between July 14, 2011, and November 22, 2012. Written informed consent was obtained from all of the participants.

A history of hypertension was prospectively recorded if patients were prescribed antihypertensive treatment or had successive blood pressure (BP) measurements that were ≥140/90 mm Hg on at least 2 different days during the index hospitalization.^23^ Noninvasive BP was measured in recumbent patients using an oscillometric method using an arm cuff pressure-sensitive transducer (GE CRITIKON, GE Healthcare, Amersham, United Kingdom) and automated medical patient monitoring system (DINAMAP and CARESCAPE Monitor B850, GE Medical Systems Information Technologies). Patients were categorized as having hypertension or not.

Patients were eligible if they had an indication for primary percutaneous coronary intervention (PCI) or thrombolysis for acute STEMI.^24^ Exclusion criteria included contraindications to CMR, for example, a pacemaker. The study was approved by the National Research Ethics Service (reference 10-S0703-28). Acute STEMI management followed contemporary guidelines^24^ (Methods in the online-only Data Supplement). The ClinicalTrials.gov identifier is URL: http://www.clinicaltrials.gov. Unique identifier: NCT02072850.

### Electrocardiogram

A 12-lead ECG was obtained before coronary reperfusion and 60 minutes afterward. The extent of ST-segment resolution on the ECG assessed 60 minutes after reperfusion compared with the baseline ECG before reperfusion^24^ was expressed as complete (≥70%), incomplete (30% to <70%), or none (≤30%).

### Coronary Angiogram Acquisition and Analyses

Coronary angiograms were acquired during usual care with cardiac catheter laboratory X-ray (Innova, GE Healthcare) and information technology equipment (Centricity, GE Healthcare). The angiograms were analyzed by trained observers (J. Carberry, V.T. Yue May) who were blinded to all other clinical and CMR data. The Thrombolysis In Myocardial Infarction (TIMI) coronary flow grade^25^ and frame count^26^ were assessed at initial angiography and at the end of the procedure. TIMI myocardial perfusion grade^27^ was assessed at the end of the procedure (Methods in the online-only Data Supplement). The TIMI frame count and perfusion grade are angiographic measures of microvascular function.

### Direct, Invasive Measurement of Microvascular Function in the Culprit Coronary Artery

A coronary pressure- and temperature-sensitive guidewire (St Jude Medical, St Paul, MN) was used to measure index of microvascular resistance (IMR) and coronary flow reserve (CFR) in the culprit coronary artery at the end of PCI.^28–32^ The guidewire was calibrated outside the body, equalized with aortic pressure at the ostium of the guide catheter and then advanced to the distal third of the culprit artery. This thermodilution method is based on the following basic relationship: flow=volume / mean transit time. CFR is defined as the ratio of peak hyperemic to resting flow (CFR=flow at hyperemia / flow at rest). Flow is the ratio of the volume (V) divided by the mean transit time (Tmn). Thus, CFR can be expressed as follows: CFR=(V/Tmn) at hyperemia/ (V/Tmn) at rest. Assuming the epicardial volume (V) remains unchanged, CFR can be calculated as follows: CFR=Tmn at rest / Tmn at hyperemia. CFR and IMR are distinct physiological parameters. CFR reflects epicardial and microcirculatory function, by contrast, IMR is a direct invasive measure of microvascular resistance. IMR is defined as the distal coronary pressure multiplied by the mean transit time of a 3 mL bolus of saline at room temperature during maximal coronary hyperemia, measured simultaneously (mm Hg×s or units).^28–32^

Hyperemia was induced by 140 μg/kg per minute of intravenous adenosine preceded by a 2 mL intracoronary bolus of 200 µg of nitrate. The mean aortic and distal coronary pressures were recorded during maximal hyperemia. We have previously found IMR to be highly repeatable when assessed by duplicate measurements 5 minutes apart in 12 consecutive STEMI patients at the end of PCI.^30^

### Laboratory Analyses

Serial systemic blood samples were obtained immediately after reperfusion in the cardiac catheterization laboratory and subsequently on the first day (06:00–07:00 hours) during the initial inpatient stay in the Coronary Care Unit.

CRP (C-reactive protein) was measured in the hospital biochemistry laboratory using a particle-enhanced immunoturbidimetric assay method (Cobas C501, Roche), and the manufacturer’s calibrators and quality control material, as a biochemical measure of inflammation. The high-sensitive assay CRP measuring range is 0.1 to 250 mg/L. The expected CRP values in a healthy adult are <5 mg/L, and the reference range in our hospital is 0 to 10 mg/L. IL (interleukin)-6 was measured using a high-sensitivity enzyme-linked immunosorbent assay (ELISA; R&D Systems, Oxon, United Kingdom).^33^ The limit of detection is <0.1 pg/mL, and the intra-assay coefficient of variation was 9.1%. NT-proBNP (N-terminal Pro-B-type natriuretic peptide) was measured in a research laboratory using an electrochemiluminescence method (e411, Roche) and the manufacturers’ calibrators and quality control material. The limit of detection for IL-6 and NT-proBNP are 6.5 pg/mL and 5 pg/mL, respectively. Long-term coefficient of variations of low and high controls are typically <5% and were all within the manufacturers’ range.

### CMR Imaging

**Figure 1.**
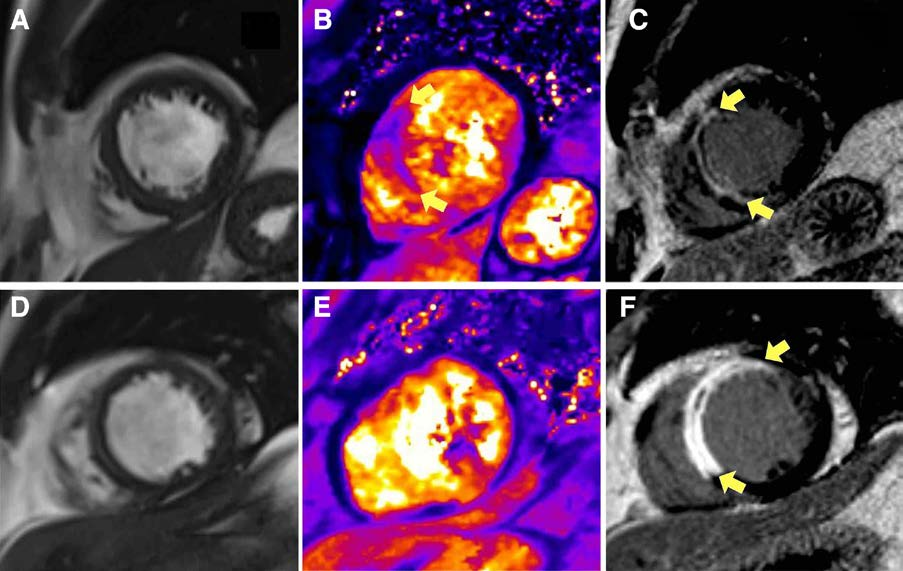
Two patients, one with a history of hypertension (**A**) and the other without (**B**) presented similarly with acute anterior ST-segment–elevation myocardial infarction and were treated by primary percutaneous coronary intervention (PCI) with stents. The antithrombotic therapies, including aspirin, clopidogrel, and unfractionated heparin, were similar. Each patient had normal antegrade flow in the culprit coronary artery (Thrombolysis In Myocardial Infarction grade 3) at the end of PCI. Multiparametric cardiovascular magnetic resonance (CMR) imaging was performed 2 d and 6 mo later. **Top**, **A**, Imaging obtained from a 52-year-old man with a history of current hypertension. The symptom-to-balloon time was 1.4 h. The coronary angiogram revealed a proximal occlusion of the left anterior descending artery. Blood pressure before coronary angioplasty was 200/125 mm Hg and measured 181/117 mm Hg postcoronary angioplasty. Two days later, CMR disclosed myocardial hemorrhage specifically revealed by T2* mapping (yellow arrows) and transmural infarction of the anteroseptal wall of the left ventricle (LV; yellow arrows) associated with microvascular obstruction revealed by contrast CMR. Invasive assessment of microvascular function using a diagnostic guidewire placed in the culprit coronary artery at the end of primary PCI indicated severe microvascular injury. The index of microvascular resistance measured 92 which is substantially increased (reference range <25). The initial infarct size was 38.9%, and the LV ejection fraction (LVEF) and LV end-diastolic volume indexed to body surface area (LVEDVi) were 48.5% and 90.2 mL/m^2^, respectively. Six months later, infarct size was 26.7% of LV mass, and the LVEDVi was 127 mL/m^2^. This is in-keeping with >20% in LVEDVi, that is, adverse remodeling. This patient went to have an unplanned admission for heart failure treatment on day 493 of follow-up. **Bottom**, **B**, Imaging obtained from a 58-year-old man with no prior history of hypertension. The symptom-to-balloon time was 2.2 h. The angiogram also revealed a proximal occlusion of the left anterior descending artery. Blood pressure before coronary angioplasty was 109/71 mm Hg and measured 99/60 mm Hg postcoronary angioplasty. Microvascular resistance in the culprit coronary artery was normal. Two days later, there was a small amount of microvascular obstruction as revealed by contrast-enhanced CMR (yellow arrows), and no evidence of myocardial hemorrhage (T2 star parametric map). The initial infarct size was 32.4%, and the LVEF and LVEDVi were 36.9% and 126.4 mL/m^2^, respectively. Six months later, infarct size was 15.2% of left ventricular mass, and the LVEDVi was 98.2 mL/m^2^. This patient had an uncomplicated clinical course.

We used CMR to provide reference data on left ventricular function, pathology, and surrogate outcomes (Figure 1). CMR was performed on a Siemens MAGNETOM Avanto (Erlangen, Germany) 1.5-Tesla scanner with a 12-element phased array cardiac surface coil.^32^ The imaging protocol^34,35^ (Methods in the online-only Data Supplement) included cine CMR with steady-state free precession, T2-mapping,^22,36,37^ T2*-mapping,^22^ and delayed-enhancement phase-sensitive inversion-recovery pulse sequences.^38^ The scan acquisitions were spatially coregistered and also included different slice orientations to enhance diagnostic confidence.

### Imaging Analyses

The CMR analyses are described in detail in the online-only Data Supplement. Left ventricular dimensions were indexed to body surface area.

#### Infarct Definition and Size

The presence of acute infarction was established based on abnormalities in cine wall motion, rest first-pass myocardial perfusion, and delayed-enhancement imaging in 2 imaging planes. The myocardial mass of late gadolinium (grams) was quantified using computer-assisted planimetry and the territory of infarction was delineated using a signal intensity threshold of >5 SDs above a remote reference region and expressed as a percentage of total left ventricular mass.^39^

#### Microvascular Obstruction

Microvascular obstruction was defined as a dark zone on early gadolinium enhancement imaging 1, 3, 5, and 7-minute postcontrast injection that remained present within an area of late gadolinium enhancement at 15 minutes.

#### Myocardial Edema

The extent of myocardial edema was defined as left ventricular myocardium with pixel values (T2) >2 SDs from remote myocardium.^40–42^

#### Myocardial Salvage

Myocardial salvage was calculated by subtraction of percent infarct size from percent area-at-risk, as reflected by the extent of edema.^40–42^ The myocardial salvage index was calculated by dividing the myocardial salvage area by the initial area-at-risk.

#### Left Ventricular Remodeling

An increase in left ventricular volume at 6 months from baseline was taken to reflect left ventricular remodeling.^35^

#### Myocardial Hemorrhage

On the T2* CMR maps, a region of reduced signal intensity within the infarcted area, with a T2* value of <20 ms^22,43^ was considered to confirm the presence of myocardial hemorrhage.

### Prespecified Health Outcomes

We prespecified adverse health outcomes that are pathophysiologically linked with STEMI.^44,45^ The primary composite outcome was (1) all-cause death or first heart failure event after the initial hospitalization (Methods in the online-only Data Supplement).

### Statistical Analyses

The sample size calculation and statistical methods are described in the online-only Data Supplement. All *P* values are 2-sided and a *P* value >0.05 indicates the absence of a statistically significant effect. Statistical analyses were performed using R version 2.15.1 or SAS v 9.3 or higher versions of these programs.

## Results

### Patient Characteristics

**Figure 2.**
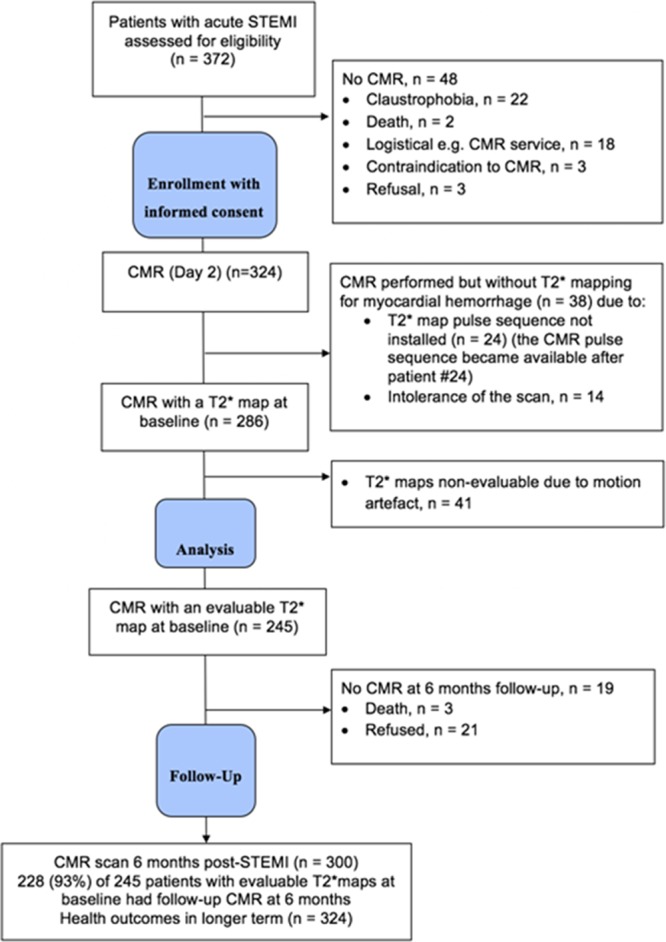
Study flow diagram of the cohort study. CMR indicates cardiovascular magnetic resonance; and STEMI, ST-segment–elevation myocardial infarction.

Of 372 patients with acute STEMI who were screened, 324 (mean age, 59 [12)] years; 237 [73%] male, 105 [32%] with hypertension) were enrolled (Table 1; Figure 2). The reasons for not being enrolled in the study are detailed in Figure 2. None of the participants had a new diagnosis of hypertension during the index admission.

**Table 1.**
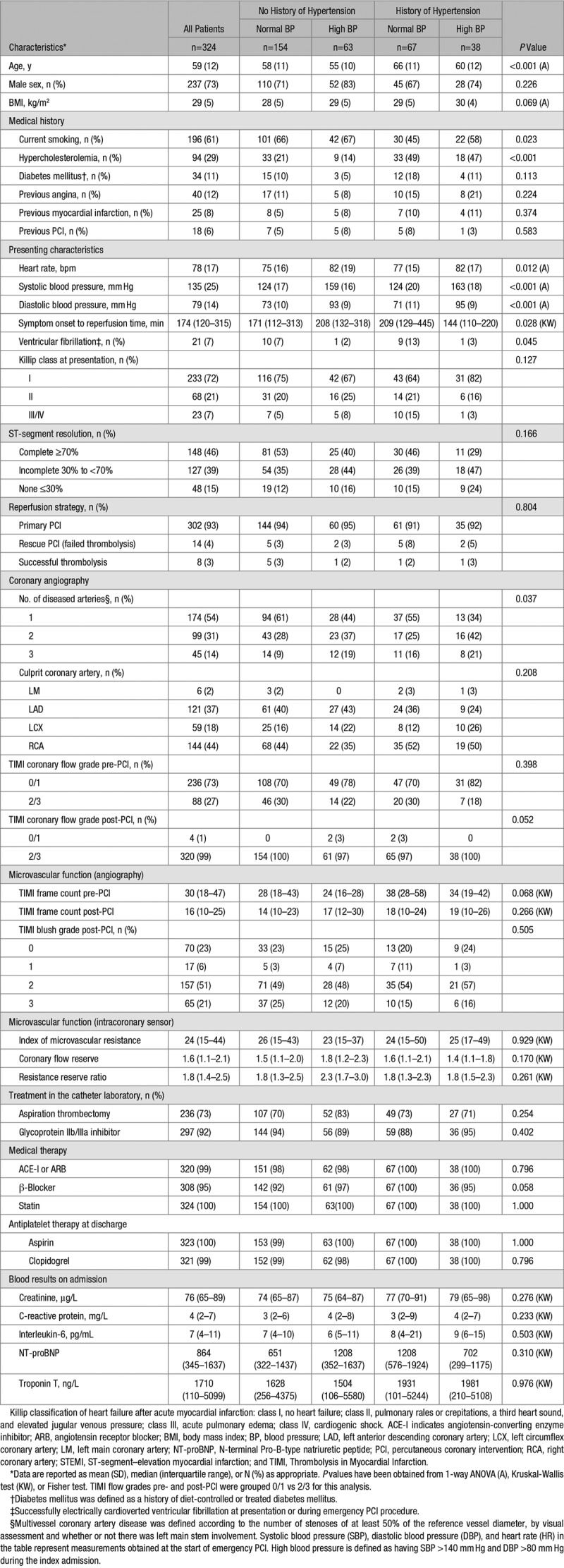
Clinical and Angiographic Characteristics of 324 STEMI Patients Categorized According to a History of Hypertension

Compared with patients without a history of hypertension, patients with a history of hypertension were older, had a history of hypercholesterolemia more often, but a history of cigarette smoking less often, and were more likely to have ventricular fibrillation at presentation and multivessel coronary artery disease (Table 1).

### Microvascular Injury in the Culprit Coronary Artery and Inflammation

Angiographic parameters of blood flow and perfusion in the culprit coronary artery, IMR, and ST-segment resolution on the ECG (none versus partial versus complete) were similar between the groups (Table 1).

On day 1, circulating CRP, IL-6, and neutrophils and monocyte levels were also similar between the groups (Table 1).

### CMR Imaging Findings

Three hundred twenty-four patients underwent CMR imaging 2.0 (1.8) days later, and 300 (93%) patients had follow-up CMR at 6 months (Table 2; Figure 2). Case examples are shown in Figure 1.

**Table 2.**
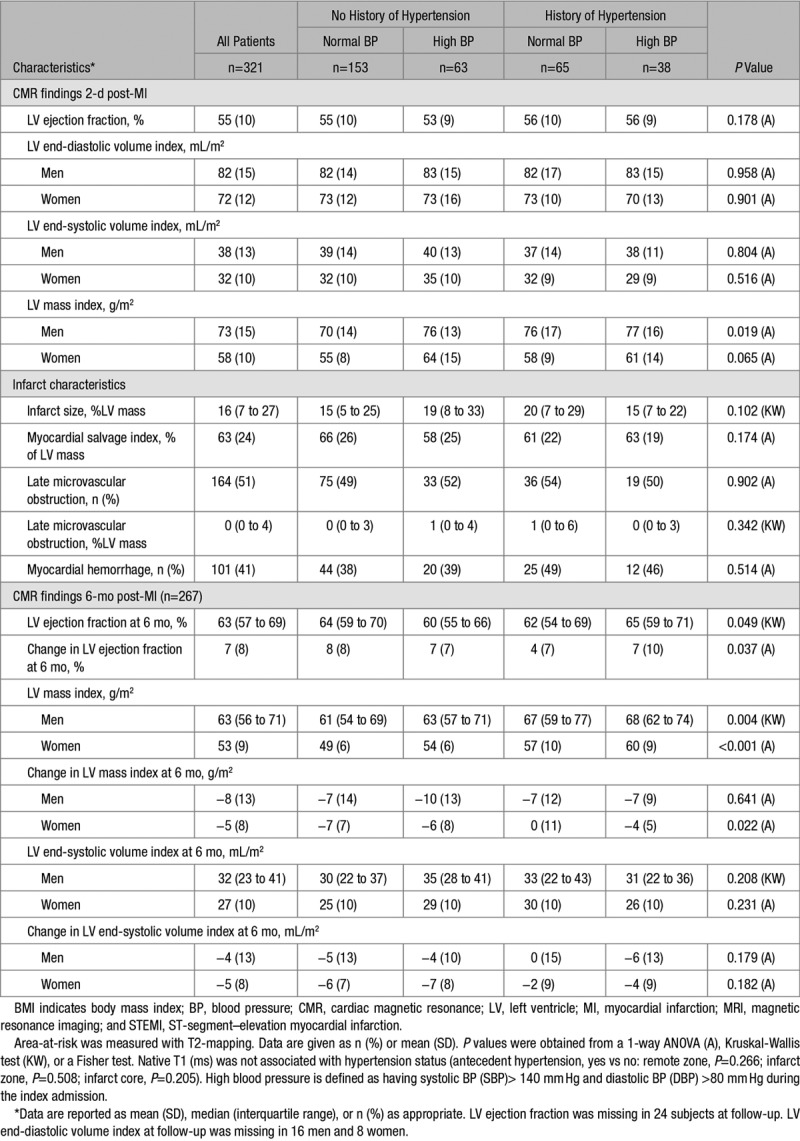
Cardiac MRI Findings at 2 Days and 6 Months Postreperfusion in 324 STEMI Patients Categorized According to History of Hypertension

Infarct size was similar in patients with or without a history of hypertension (Table 2). However, left ventricular mass index at baseline was associated with a history of hypertension in men (Table 2) and in both men and women with hypertension at 6-month post-STEMI (Table 2).

Left ventricular ejection fraction improved in all patients, however, compared with patients without a history of hypertension, the increase in ejection fraction was less in patients with a history of hypertension (Table 2).

#### Sex Differences and History of Hypertension

Left ventricular mass reduced to a lesser extent by 6 months in women with hypertension compared with in women without hypertension (Table 2).

### BP at Initial Presentation

We observed associations between BP at the start of emergency PCI (normal BP [systolic BP ≤140 mm Hg; diastolic BP ≤80 mm Hg]; high BP [systolic BP >140 mm Hg; diastolic BP >80 mm Hg] and clinical characteristics notably body mass index [normal BP versus high BP: 28.4 (4.8) versus 29.6 (4.7) kg/m^2^; *P*=0.042] but not vascular risk factors [smoking *P*=0.54]; hypercholesterolemia [*P*=0.59]) and microvascular dysfunction at the end of PCI as revealed by ST-segment resolution (complete, none, partial: [normal BP versus high BP] 111 [50.5%], 29 [13.2%], 80 [36.4%] versus 36 [35.6%], 19 [18.8%], 46 [45.5%]; *P*=0.041).

### Multivariable Associations Between Hypertension and Coronary Microvascular Pathology

#### Myocardial Hemorrhage

In a binary logistic regression model with baseline characteristics, a history of hypertension was a multivariable associate of myocardial hemorrhage (odds ratio, 1.81; [95% confidence interval, 0.98–53.34]; *P*=0.059; Table 3), albeit with wide confidence intervals.

**Table 3.**
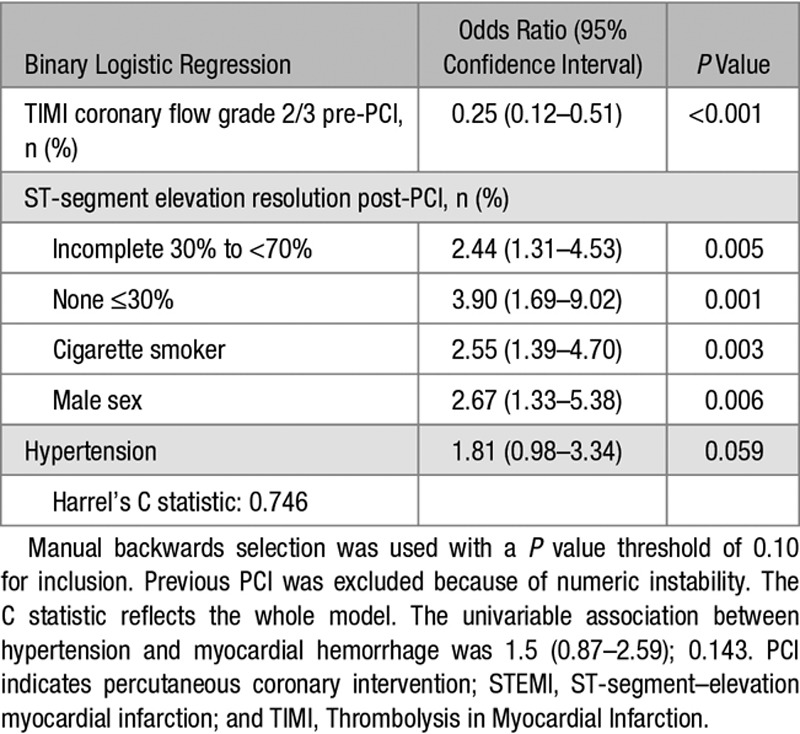
Multivariable Binary Logistic Regression Model of the Associations Between Clinical Characteristics, Including a History of Hypertension (Present or Absent), and the Occurrence of Myocardial Hemorrhage (Yes or No) 2 Days Later (n=324) in Patients With Acute STEMI

### Microvascular Dysfunction and Health Outcomes in the Longer Term

All (n=324) of the patients had long-term follow-up data completed. The median duration of follow-up was 1500 days (postdischarge censor duration [range] 1236 to 1801 days). Forty-seven (15%) patients died or experienced a first heart failure event during the index hospitalization or postdischarge. These events included 4 cardiovascular deaths, 11 noncardiovascular deaths, 2 deaths of undetermined cause, and 30 episodes of heart failure (Killip class 3 or 4 heart failure [n=28] or defibrillator implantation n=2). Twenty-three (7%) patients died or experienced a first heart failure hospitalization postdischarge.

A history of hypertension (odds ratio, 2.53; [95% confidence interval, 1.28–4.98]; *P*=0.007) was a multivariable associate of all-cause death or heart failure (Table 4).

**Table 4.**
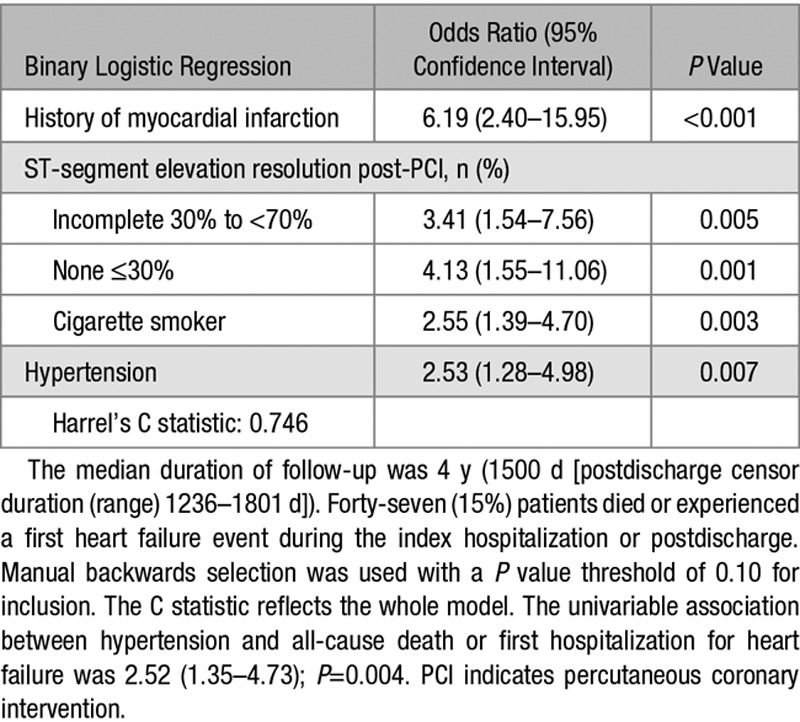
Multivariable Binary Logistic Regression Model for the Composite End Point (Yes or No) of All-Cause Death or First Hospitalization for Heart Failure, Including Clinical Characteristics (Present or Absent) at Baseline

## Discussion

We have undertaken a large prospective imaging cohort study of hypertension status, microvascular pathophysiology, and long-term prognosis in patients with an acute STEMI. Uniquely, our study enrolled a high proportion of screened patients (nearly 9 of every 10 assessed), followed by serial multimodality assessments including use of invasive and noninvasive tests of reperfusion injury, circulating measures of inflammation, serial imaging of infarct pathology and remodeling, and follow-up for health outcomes over a median of 4 years in all of the study participants.

The main findings are that antecedent hypertension was associated with (1) older age and less cigarette smoking; (2) a 2-fold increased likelihood of myocardial hemorrhage, albeit with wide confidence intervals, and less improvement in left ventricular systolic function at 6 months; (3) sex differences, specifically, for the associations between hypertension status and left ventricular outcomes in women but not in men; and (4) >2-fold increased risk of all-cause death or heart failure during a median of 4 years follow-up. Antecedent hypertension was not associated with infarct size, reperfusion injury, or systemic inflammation. BP status at initial presentation was associated with age, body mass index, and reperfusion injury as revealed by ST-segment resolution at the end of the PCI procedure.

### Hypertension and Prognosis After Acute STEMI

In line with prior studies,^11,12^ we found that a history of hypertension is associated with risk factors for cardiovascular disease including age, smoking (less common),^46^ and hypercholesterolemia (more common).

We also found that a history of hypertension was independently associated with an increased risk of all-cause death or hospitalization for heart failure. This result extends the evidence from previous studies. Notably, Reinstadler et al^11^ found that antecedent hypertension was associated with a >3-fold risk of major adverse cardiac events at 12 months in a clinical trial population of 792 patients with acute STEMI.

### Antecedent Hypertension, Sex, and Remodeling

Recent studies have reported conflicting information on the associations between antecedent hypertension and sex.^11,12^ Changes in left ventricular ejection fraction and remodeling at 6 months, including left ventricular end-systolic volume and mass, were less favorable in women with hypertension compared with women without hypertension. The enhanced left ventricular mass in women with hypertension post-MI predisposes these individuals to adverse remodeling post-MI, and potentially, a worse cardiac prognosis in the longer term. Since the mean age of the participants was 59 years, an accelerated cardiovascular risk in postmenopausal women may be one contributing factor. Although reductions in mortality attributable to coronary heart disease have been observed in recent decades, no such decline has been observed in younger (<55 years) women.^47^ Further research seems warranted.

### Antecedent Hypertension, Microvascular Function, and Myocardial Hemorrhage Post-MI

We studied the relationships between microvascular resistance measured directly in the culprit coronary artery at the time of the acute STEMI and antecedent hypertension. Surprisingly, hypertension was not associated with acute reperfusion injury, as revealed by direct intracoronary measurements of microvascular resistance and by angiographic parameters (TIMI frame count, TIMI blush grade) or ST-segment resolution. The potential explanations for this finding include the prior use of antihypertensive therapies, such as angiotensin-converting enzyme inhibitors, which have protective effects on vascular function,^23^ and the similar levels of arterial BP at the time of hospital admission in patients with a history of hypertension compared with BP levels in patients with no history of hypertension. This finding is in-keeping with the beneficial effects of both lifestyle and pharmacological measures to control BP. Because all of these parameters of coronary microvascular function are associated with prognosis post-MI,^26,27,32^ our findings rule out enhanced microvascular injury within the infarct zone as an explanation for the adverse prognosis in patients with antecedent hypertension.

Myocardial hemorrhage occurs in about one-third of patients with acute STEMI.^21,22^ This pathology reflects the end-stage consequence of irreversible microvascular dysfunction and is independently associated with adverse cardiac outcomes.^21,22^ In a time-course study,^48^ we have previously shown that myocardial hemorrhage occurs in 2 phases after coronary reperfusion. The first phase occurs acutely within 12 hours. The second phase involves new, secondary bleeds that occur between days 1 and 3 in previously unaffected patients.^48^ In this study, all patients who had evidence of new myocardial hemorrhage on day 3 had prior evidence of microvascular obstruction at 12 hours. We think that the temporal relationships between microvascular obstruction and myocardial hemorrhage may be relevant when considering their associations with hypertension status.

Microvascular function measured acutely and microvascular obstruction revealed by CMR 2 days later were not associated with hypertension status. However, myocardial hemorrhage, as specifically revealed by T2* mapping (Figure 1) was associated with a near 2-fold increased risk of hypertension, independent of other predictors. The result was not statistically significant and thus hypothesis generating. Cigarette smoking status is a multivariable, positive associate of myocardial hemorrhage after an acute STEMI (Table 3)^48^ and the inverse association between hypertension and smoking status may be a relevant confounding factor.

We undertook a time-course study with repeated assessments to assess the temporal evolution of microvascular injury acutely and then subsequently 2 to 3 days later using CMR. Long-term follow-up of this cohort permitted an analysis of the prognostic significance of microvascular injury early post-MI. Myocardial hemorrhage reflects vascular degradation and capillary leak of red blood cells. Hemorrhage within the infarct zone as revealed by CMR (Figure 1) is a direct measure of end-stage vascular injury post-MI. Our findings lead to a conclusion that despite a similar extent of acute microvascular injury and infarct size, vascular degradation at 2 days is greater in patients with a history of hypertension compared with in patients with no prior hypertension. We hypothesize that the microvessels of patients with hypertension are less capable of maintaining vascular integrity under conditions of ischemia/reperfusion injury.^49^ Given that hemorrhage may develop progressively in a secondary phase (days 2–3), impaired vascular homeostasis and repair potential in patients with chronic hypertension may be explanations for these results. Such patients may have preexisting coronary microvascular disease,^15,16^ and the microvessels subtended by the culprit artery may be less resistant to the effects of reperfusion injury (acidosis, oxidants, etc), leading to progressive capillary degradation and infarct zone hemorrhage. A susceptibility to hemorrhagic transformation within the infarct zone may provide a new mechanistic explanation for why patients with antecedent hypertension have a worse prognosis despite infarct size being similar to patients without prior hypertension.^11,12^ Accumulation of deoxyhemoglobin and iron within the infarct zone may serve as a mechanistic substrate for enhanced scar formation and abnormal left ventricular remodeling.^50^ Our results suggest that progressive microvascular damage within the infarct zone in patients with antecedent hypertension may underpin an impaired recovery potential within the heart, leading in turn to left ventricular systolic dysfunction and heart failure in the longer term.

We did not find any association between hypertension status and infarct size, as reflected by contrast-enhanced CMR and peak troponin concentration. This result is consistent with reports by Reinstadler et al^11^ and De Luca et al.^12^ We did not observe any association between hypertension status and circulating measures of inflammation. This result could potentially be explained by the anti-inflammatory effects of antihypertensive drug therapies, such as angiotensin-converting enzyme inhibitors.

Our results provide further evidence that a history of hypertension in patients with an acute STEMI is associated with a worse prognosis. In terms of clinical translation, our results highlight that patients with a history of hypertension are at an increased risk of developing heart failure. The results support further research into therapeutic strategies designed to preserve vascular integrity and repair potential within the vascular distribution of the culprit coronary artery.

### Limitations

Our analysis does not permit inference on causality, and further studies are warranted. We lacked detailed information on BP history and compliance with antihypertensive drug therapy before the index hospitalization.

### Perspectives

In summary, we have studied the complex relationships between hypertension status, concomitant risk factors, infarct pathology, left ventricular remodeling, and health outcomes in a large cohort of STEMI patients. We found that a history of hypertension in patients with acute STEMI is independently associated less improvement in left ventricular systolic function, notably in women, and an increased risk of all-cause death and heart failure in the longer term. An increased propensity to myocardial hemorrhage may be one mechanistic explanation, reflecting severe microvascular injury.

Our results confirm and extend previous investigations and support further research into therapeutic strategies that attenuate reperfusion injury within the infarct zone in patients with acute STEMI.

## Acknowledgments

We thank the patients who participated in this study and the staff in the Cardiology and Radiology Departments. We thank Peter Weale and Patrick Revell (Siemens Healthcare, United Kingdom).

## Sources of Funding

This study was supported by the British Heart Foundation (BHF) Centre of Research Excellence Award (RE/13/5/30177), the BHF Project Grant PG/11/2/28474, the National Health Service, and the Chief Scientist Office. C. Berry was supported by a Senior Fellowship from the Scottish Funding Council. P. Welsh is supported by BHF Fellowship FS/12/62/29889. A.M. Maznyczka is supported by BHF Fellowship FS/16/74/32573.

## Disclosures

Based on institutional agreements with the University of Glasgow, Siemens Healthcare has provided work-in-progress imaging methods and C. Berry has acted as a consultant to Abbott Vascular. K.G. Oldroyd has acted as consultant to Abbott Vascular and Volcano Corporation. These companies had no involvement in the current research or the article. The other authors report no conflicts.

## Supplementary Material

## Novelty and Significance

### What Is New?

- The pathological and prognostic significance of a history of hypertension was assessed in a reasonably large cohort of patients with acute ST-segment–elevation myocardial infarction treated by emergency percutaneous coronary intervention.
- Hypertension was associated with a near 2-fold increased likelihood of myocardial hemorrhage, reflecting severe microvascular injury.
- Hypertension was associated with less favorable changes in left ventricular ejection fraction and remodeling, notably in women, and hypertension was associated with a higher risk of all-cause death and heart failure.

### What Is Relevant?

- A history of hypertension is associated with a worse prognosis.
- Patients with acute ST-segment–elevation myocardial infarction and a history of hypertension merit intensive medical management.

### Summary

Our results confirm and extend previous investigations and support further research into therapeutic strategies that attenuate reperfusion injury within the infarct zone in patients with acute ST-segment–elevation myocardial infarction.
